# The Interplay between Oxidative Stress and Sphingolipid Metabolism in Endometrial Cancer

**DOI:** 10.3390/ijms251910243

**Published:** 2024-09-24

**Authors:** Agnieszka U. Błachnio-Zabielska, Patrycja Sadowska, Michał Zdrodowski, Piotr Laudański, Jacek Szamatowicz, Mariusz Kuźmicki

**Affiliations:** 1 Department of Hygiene, Epidemiology and Metabolic Disorders, Medical University of Bialystok, 15-089 Bialystok, Poland; agnieszka.blachnio@umb.edu.pl (A.U.B.-Z.); patrycja.sadowska@umb.edu.pl (P.S.); 2 Department of Gynecology and Gynecological Oncology, Medical University of Bialystok, 15-276 Bialystok, Poland; michal.zdrodowski@umb.edu.pl (M.Z.); jacek.szamatowicz@umb.edu.pl (J.S.); 3 Department of Obstetrics, Gynecology and Gynecological Oncology, Medical University of Warsaw, 02-091 Warsaw, Poland; piotr.laudanski@wum.edu.pl; 4 Women’s Health Research Institute, Calisia University, 62-800 Kalisz, Poland; 5 OVIklinika Infertility Center, 01-377 Warsaw, Poland

**Keywords:** endometrial cancer, sphingolipids, oxidative status, mass spectrometry

## Abstract

Endometrial cancer is one of the most common malignancies in women. Sphingolipids, a group of lipids, play a key role in cancer biology. Cancer cells often exhibit abnormal redox homeostasis characterized by elevated levels of reactive oxygen species (ROS). Emerging evidence suggests that ceramides are involved in inhibiting proliferation and inducing apoptosis through ROS production. However, there is no data on the relationship between sphingolipid metabolism and oxidative status in endometrial cancer. The present study aims to assess the content of individual sphingolipids and oxidative status in healthy women and those with endometrial cancer. Sphingolipid analysis was performed using mass spectrometry. Total oxidative status (TOS) and total antioxidant capacity (TAC) were assessed colorimetrically. Our results showed a significant increase in the levels of all measured sphingolipids in cancer tissues compared to healthy endometrium. Additionally, a significant decrease in the S1P/ceramide ratio (sphingolipid rheostat) was observed in cancer patients, particularly for C14:0-Cer, C16:0-Cer, C18:1-Cer, C22:0-Cer, and C24:0-Cer. Furthermore, increased TOS and decreased TAC were found in cancer patients compared to healthy women. Significant correlations were observed between the levels of individual sphingolipids and oxidative status, with the strongest correlation noted between C22:0-Cer and TOS (r = 0.64). We conclude that endometrial cancer is characterized by profound changes in sphingolipid metabolism, contributing to oxidative dysregulation and tumor progression.

## 1. Introduction

The incidence of endometrial cancer cases is rapidly increasing in developed countries, mainly due to the growing number of obese patients, the use of tamoxifen in breast cancer treatment, and increasing lifespan [1]. Diagnosis of the disease in stage I gives a relatively good prognosis, however, if the disease continues to spread, the patient survival rate dramatically decreases [2].

The research on metabolic changes in cancer cells conducted in the first half of the 20th century has led to the identification of two main subtypes of endometrial cancer, differing in etiology, aggressiveness, and the age at which they develop. Type I, accounting for approximately 80% of cases, is endometrioid carcinoma that is estrogen-dependent [3]. In this type, estrogen signaling via the estrogen receptor acts as an oncogenic signal. It most commonly develops around the menopausal age [4]. Type II endometrial cancer, much rarer, affects women in the 6th–8th decade of life. This type includes serous and clear cell adenocarcinoma and, unlike type I, it is estrogen-independent, and most often exhibits a high degree of malignancy. Clinical data indicate that type I accounts for the majority of cases of endometrial cancer, with approximately 40% being associated with obesity. Obesity is a condition characterized by excessive accumulation of adipose tissue in the body, accompanied by hormonal, carbohydrate, and lipid metabolism disorders [5]. Sphingolipids constitute an important group of biologically active lipids, the metabolism of which changes in the state of obesity. These compounds play a significant role in regulating cellular processes such as proliferation, cell differentiation, apoptosis, growth inhibition, inflammatory response, and angiogenesis [6]. The central compound in sphingolipid metabolism is ceramide, that exhibits pro-apoptotic effects, and a growing body of evidence suggests that ceramides are involved in cancer development and progression [7].

**Figure 1:**
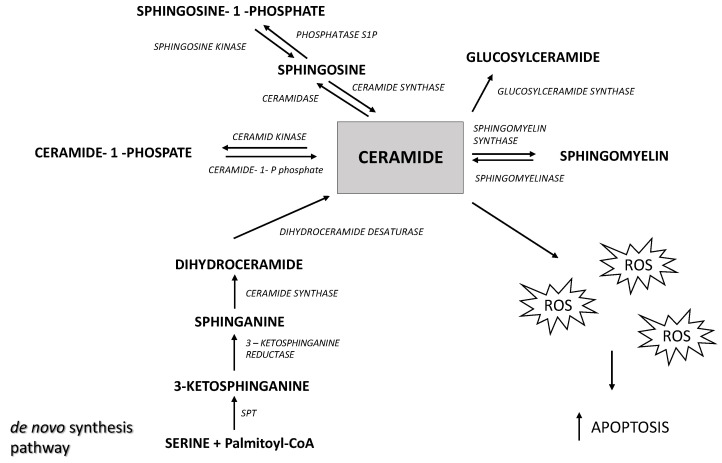
The overview of sphingolipid metabolism. SPT—serine palmitoyltransferase, ROS—reactive oxygen species.

The level of ceramide in the cell depends on the rate of its formation and degradation (Figure 1). The main sources of ceramides in the cell are de novo synthesis, that occurs in the endoplasmic reticulum, and sphingomyelin hydrolysis catalyzed by sphingomyelinases.

Ceramide undergoes further metabolic transformations into sphingosine (Sph) in a reaction catalyzed by ceramidases. Subsequently, Sph is phosphorylated by sphingosine kinase, leading to the formation of sphingosine-1-phosphate (S1P) [8]. It should be emphasized that ceramide and S1P play opposing roles in oncogenesis; ceramide is a pro-apoptotic compound whereas S1P is a pro-proliferative compound, and the S1P/Cer ratio (described as a sphingolipid rheostat) determines whether cells are directed towards proliferation or apoptosis [9]. Ceramides and S1P are also involved in regulating the process of mitophagy, during which damaged mitochondria are encased in double-membrane vesicles (called autophagosomes) for degradation [10]. Lethal mitophagy is an anti-cancer mechanism mediated by ceramide, during which cells degrade numerous mitochondria, leading to cancer cell death in an apoptosis-independent manner [10]. The increase in sphingolipid levels may reflect alterations in cellular signaling and membrane dynamics that support tumor growth and survival. Previous studies suggest that a reduced ceramide content may lead to the excessive proliferation of cancer cells [11]. On the other hand, elevated S1P levels may promote angiogenesis and cancer cell invasion. Growing evidence indicates that the anti-proliferative and pro-apoptotic effects of ceramide may occur through the stimulation of reactive oxygen species (ROS) production [12,13,14,15]. Excess ROS disturb the cellular oxidative balance, which ultimately leads to cell death. Cancer cells often exhibit abnormal redox homeostasis characterized by elevated cellular ROS levels [16]. However, depending on the concentration, ROS affect tumor progression by enhancing cell proliferation, but at very high levels they exhibit cytotoxic effects and induce cell death [17]. One of the most significant effects of oxidative stress is lipid peroxidation—the oxidation of membrane lipids by reactive oxygen species, which plays a crucial role in the regulation of apoptosis. Lipid peroxidation products, such as malondialdehyde (MDA) and 4-hydroxynonenal (4-HNE), can act as signals triggering apoptosis by activating various signaling pathways [18]. Lipid peroxidation causes damage to cell membranes, leading to the loss of mitochondrial membrane integrity and the release of pro-apoptotic proteins, such as cytochrome c. Additionally, lipid peroxidation products can modify proteins and DNA, inducing cellular stress and activating caspases—enzymes that are critical to the apoptosis process [18].

There are no data in the literature regarding sphingolipid metabolism and oxidative stress in endometrial cancer. Therefore, the aim of this study was to answer the question concerning the role of sphingolipids in relation to oxidative stress in the pathogenesis and development of endometrioid carcinoma.

## 2. Results

### 2.1. Characteristics of the Studied Groups

The characteristics of both groups are presented in Table 1. The patients in both groups were matched for age, body weight, BMI, and fasting glucose concentration. However, the OGTT revealed significantly higher glucose concentrations at 60 min and 120 min after glucose loading in the EC group as compared to the control group. The average age in the control group was 59 years (ranging from 36 to 82 years), while in the EC group it was 64 years (ranging from 39 to 86 years). The average weight in the control group was 80 kg (ranging from 72 to 116 kg) whereas in the EC group it was 83 kg (ranging from 50 to 139 kg). The average BMI in the control group was 29.76 kg/m^2^ (ranging from 20.9 to 39.21 kg/m^2^), and in the EC group it was 32.23 kg/m^2^ (ranging from 21.64 to 54.98 kg/m^2^). The average fasting glucose concentration in the control group was 108.13 mg/dL (ranging from 81 to 143 mg/dL), while in the EC group it was 116.97 mg/dL (ranging from 84 to 231 mg/dL). The mean HOMA-IR value in the control group was 8.89 (ranging from 0.93 to 18.07) whereas in the EC group it was 18.30 (ranging from 0.96 to 48.87). Despite the substantially higher HOMA-IR value in the EC group as compared to the control group, this difference was not statistically significant. The average total cholesterol concentration was 208.8 mmol/L in the control group and 176.23 mmol/in the EC group, showing statistical significance (*p* = 0.0303). The HDL cholesterol concentration was 60.40 mmol/L (ranging from 34.41 to 106.93 mmol/L) in the control group and 56.82 mmol/L (ranging from 26.62 to 83.74 mmol/L) in the EC group. The LDL cholesterol concentration was 128.90 mmol/L (ranging from 56.6 to 217.8 mmol/L) in the control group and 108.72 mmol/L (ranging from 73.2 to 152.3 mmol/L) in the EC group. The triglyceride concentration was 145.15 mmol/L (ranging from 60 to 280 mmol/L) in the control group and 122 mmol/L (ranging from 41 to 267 mmol/L) in the EC group.

### 2.2. Sphingolipids

The content of all ceramides in tissue was significantly higher in women with endometrial cancer as compared to the endometrium of healthy women (*p* < 0.0001). The content of all the analyzed sphingolipids was higher in cancerous tissue as compared to the endometrium of the control group. The concentrations of individual sphingolipids in both groups are presented in Table 2.

No statistically significant differences were observed in serum sphingolipid concentrations between the group of women with endometrial cancer and healthy women. The concentrations of individual sphingolipids in serum for both groups are presented in Table 3.

**Figure 2:**
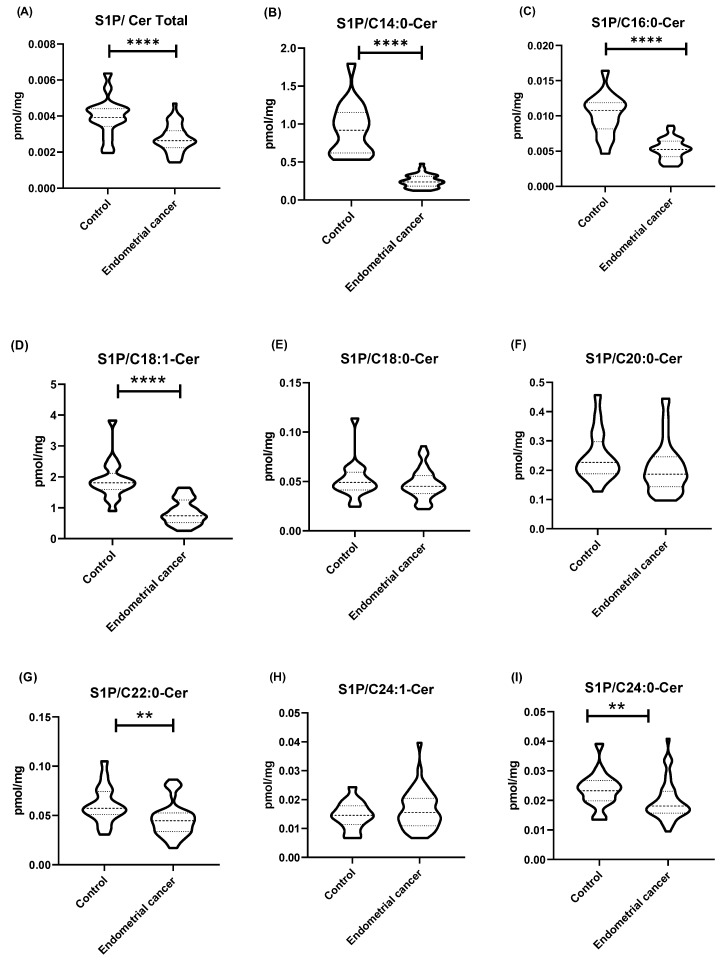
(**A**–**I**) Sphingolipid rheostat—the ratio of S1P concentration (pmol/mg tissue) to ceramide concentration (pmol/mg tissue). Data are presented as medians (interquartile range) and the differences between groups were compared by the Mann–Whitney U test; ** *p* < 0.01, **** *p* < 0.0001 indicate significant differences from the controls.

The values of the sphingolipid rheostat (the ratio of S1P concentration to ceramide concentration) in tissues for most ceramides (C14:0-Cer, C16:0-Cer, C18:1-Cer, C22:0-Cer, C24:0-Cer, and total Cer) were significantly lower in the endometrial cancer group as compared to the control group (*p* < 0.0001). No significant differences in the sphingolipid rheostat were observed between the control group and the EC group for C18:0-Cer and C20:0-Cer (*p* > 0.05). The sphingolipid rheostat in the control tissues and in the group of endometrial cancer patients are presented in Figure 2.

### 2.3. Redox Homeostasis and Lipid Peroxidation

**Figure 3:**
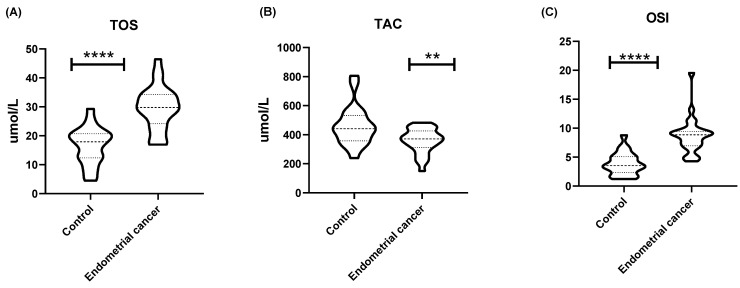
(**A**) The value of total oxidative status (TOS) (µmol/L), (**B**) total antioxidant capacity (TAC) (µmol/L), and (**C**) OSI expressed as [19]: [TOS]/[TAC] × 100%. Data are presented as medians (interquartile range). Analysis was performed with the Mann–Whitney U test; ** *p* < 0.01, **** *p* < 0.0001 indicate significant differences from the controls.

The total oxidant status (TOS) in endometrial cancer tissues was significantly higher than in the endometrium of healthy women (*p* < 0.0001). On the other hand, the total antioxidant capacity (TAC) was significantly lower in endometrial cancer as compared to the endometrium of the control group of women (*p* < 0.01). Finally, the oxidative stress index (OSI) was significantly higher in cancerous tissues as compared to healthy endometrial tissues (*p* < 0.0001) (Figure 3). MDA levels in endometrial cancer were significantly higher (*p* < 0.0001) than in healthy endometrial tissue (Table 1).

The results of the comparative analysis of both groups of women indicated statistically significant differences between the control group and the group of patients with endometrial cancer in terms of oxidative stress. The patients with endometrial cancer (EC) had higher TOS (*p* ≤ 0.0001) and OSI (*p* ≤ 0.0001) values, which indicated a greater amount of oxidizing compounds in the study group. On the other hand, the TAC value was significantly lower in the EC group as compared to the control group of women (*p* = 0.006), which indicated reduced antioxidant capacity.

### 2.4. Correlations between Oxidative Status and Lipid Concentration

The conducted Pearson correlation analysis showed the existence of statistically significant correlations between TOS and Sph (r = 0.507), SPA (r = 0.557), C20:0-Cer (r = 0.501), C22:0-Cer (r = 0.641), C24: 0-Cer (r = 0.551), and total Cer (r = 0.521). A statistically significant correlation was also found between OSI and SPA (r = 0.542), C16:0-Cer (r = 0.493), C22:0-Cer (r = 0.582), C24:0-Cer (r= 0.508), and total Cer (r= 0.522) (Table 4). 

## 3. Discussion

Our study highlights the significant association between metabolic disturbances of sphingolipids and oxidative stress in type I endometrial cancer, which accounts for approximately 80% of all endometrial cancer cases [3]. This study is the first to investigate the interconnection between sphingolipid profile and oxidative stress. The results of our work reveal significant differences in lipid metabolism and oxidative stress between women with type I endometrial cancer and the healthy control group. There is a well-documented association between a higher BMI and an increased risk of endometrial cancer, likely due to the role of adiposity in promoting inflammation and hormonal dysregulation [5]. Increased adiposity in obese individuals leads to elevated estrogen levels through increased aromatase activity [20]. Furthermore, obesity disrupts lipid metabolism and is linked to increased lipid production, including sphingolipids [21]. However, in our study, both groups of women were matched for age and BMI. Although both groups of patients had similarly elevated BMIs, we observed a significant increase in sphingolipid levels in the endometrial cancer group as compared to the control group, indicating a distinct mechanism regulating lipid metabolism in endometrial cancer. This hypothesis is supported by metabolomic studies that have shown the most significant differences between healthy women and those with endometrial cancer, pertaining to lipid profiles [22,23,24,25]. In our study, one of the most noteworthy findings has arisen from the elevated levels of all the measured sphingolipids in cancer tissues as compared to healthy endometrial tissues. Sphingolipids as bioactive molecules are involved in regulating important cellular processes such as proliferation, apoptosis, and inflammation, that are crucial in cancer pathogenesis, invasion, and metastasis [26]. The increase in ceramide levels in cancer tissues observed in our work is consistent with existing studies that have identified altered sphingolipid metabolism as a characteristic feature of various cancers, including endometrial cancer [26,27,28,29]. However, the data available in the related literature are not unequivocal, and some of them indicate that in many types of cancer, the level of ceramides is decreased. For example, a decrease in ceramide content has been observed in aggressive glioblastoma multiforme [30,31,32,33], colon cancer [34], human astrocytoma [33], and ovarian carcinoma [35]. Interestingly, Moro et al. [36] have demonstrated that in breast cancer, ceramide levels are higher in estrogen-positive cancer as compared to estrogen-negative cancer and have postulated that elevated sphingolipid levels are associated with lower malignancy. Similarly, our study has shown increased levels of all the sphingolipids in type I endometrial cancer, which is estrogen-positive and significantly less aggressive than type II endometrial cancer. That correlation suggests a potential role of sphingolipids in modulating tumor aggressiveness. One possible mechanism contributing to the reduced ability of tumors with increased ceramide content to progress is the ability of ceramides to inhibit the expression of matrix metalloproteinases (MMPs). MMPs are proteolytic enzymes that play a key role in tumor invasion and metastasis [37]. Moreover, another important possible effect of elevated ceramide levels in cancer cells is an increase in oxidative stress, which promotes cancer cell death induced by the ROS excess.

However, the emerging evidence indicates that not all ceramides play the same role in the cell, and ceramides with varying fatty acid chain lengths may play diverse roles in regulating the growth and death of cancer cells. The available data indicate that ceramides synthesized de novo, with the involvement of various ceramide synthases (CerS), may play opposing roles in the regulation of processes related to cell death or survival. Senkal et al. have demonstrated that in head and neck squamous cell carcinoma, C18-Cer, synthesized with the involvement of CerS1, plays a pro-apoptotic role whereas C16-Cer, synthesized by CerS6, plays an anti-apoptotic role [38,39,40]. Moreover, the available data suggest that ceramides with varying fatty acid chain lengths may have a variety of functions not only in the regulation of tumor growth but also in response to treatment. It has been found that C18-Cer sensitizes cancer cells to several chemotherapy drugs, including cisplatin, gemcitabine, doxorubicin, and vincristine [38,41]. In our study, we have observed an increase in all the analyzed sphingolipids in endometrial cancer. However, we do not know whether individual ceramides play the same role in endometrial cancer as they do in head and neck squamous cell carcinoma.

A growing body of evidence suggests that, in the regulation of cellular processes related to survival or cell death, the sphingolipid rheostat—the balance between S1P and ceramides—plays a crucial role in determining cell fate rather than the absolute level of ceramide [42]. Dysregulation of sphingolipid metabolism, manifested by a change in the sphingolipid rheostat, determines cell death or survival [42]. Sphingolipids, including ceramide and S1P, may act as signals regulating cell survival or death, depending on their relative concentrations and cellular contexts [43]. Understanding and modulating this balance could have significant therapeutic implications. Our data have shown the S1P/total ceramide rheostat to be significantly lower in the cancer group as compared to the control group. The higher S1P/ceramide ratio generally promotes cell survival and proliferation, while a lower ratio favors apoptosis. The observed decrease in the rheostat values, including specific ceramide species (C14-Cer, C16-Cer, C18:1-Cer, C22-Cer, and C24-Cer), suggests a shift towards a pro-apoptotic state, which may reflect a cellular response to the tumor microenvironment or dysregulated ceramide metabolism in cancer cells. Interestingly, the related literature data suggest that the aggressiveness of cancer may also be determined by the sphingolipid rheostat rather than the absolute ceramide levels. For example, in gliomas with a mutation in the isocitrate dehydrogenase (IDH) gene, that are characterized by lower malignancy [44], reduced activity of sphingosine kinase 1 has been demonstrated, resulting in decreased S1P levels [30]. The reduction in S1P levels shifts the sphingolipid rheostat to lower values, thereby promoting apoptosis in this type of glioma. 

Alterations in sphingolipid metabolism leading to increased ceramide levels are often associated with enhanced ROS production and the induction of oxidative stress. Oxidative stress is known to contribute to carcinogenesis by inducing DNA damage, promoting genetic instability, and supporting a pro-inflammatory environment [45,46,47]. Our study has revealed significant changes in oxidative stress markers in cancer patients. The total oxidative status was significantly higher in the endometrial cancer group, indicating increased oxidative stress. Additionally, we observed a significant increase in lipid peroxidation, as evidenced by elevated MDA levels in endometrial cancer tissue compared to healthy endometrium in the control group, further confirming heightened oxidative stress in endometrial cancer. These findings are consistent with previous studies that have also reported increased lipid peroxidation in endometrial cancer [48]. Conversely, the total antioxidant capacity was reduced, suggesting a compromised antioxidant defense system in those patients. These results are consistent with previous studies that have shown increased oxidative stress and reduced antioxidant capacity in cancer patients [45]. That imbalance is further corroborated by the oxidative stress index, which was significantly elevated in the cancer group. 

In our study, we have also identified several significant correlations between oxidative stress markers and sphingolipid levels. Both TOS and OSI have correlated most strongly with the content of C22:0-Cer in endometrial tissues (r = 0.64 and r = 0.58, respectively). These correlations suggest that increased oxidative stress is associated with higher levels of specific sphingolipids, indicating a potential feedback mechanism in which oxidative stress influences lipid metabolism in endometrial cancer. These findings suggest a close interdependence between oxidative stress and sphingolipid metabolism in endometrial cancer, consistent with previous reports linking oxidative stress to altered lipid profiles in cancer cells [49]. Furthermore, the significant role of C22:0-Cer in the pathogenesis of endometrial cancer has been demonstrated in metabolomic studies that have shown that plasma ceramides, particularly C22:0-Cer, may have potential diagnostic value and could be used as biomarkers for endometrial cancer [50].

In summary, our study demonstrates a clear link between altered sphingolipid metabolism, increased oxidative stress, and endometrial cancer. These findings provide insights into the biochemical alterations associated with endometrial cancer and suggest potential therapeutic targets. Future research should focus on elucidating the mechanisms driving these changes and exploring the therapeutic potential of modulating sphingolipid and oxidative stress pathways in endometrial cancer.

## 4. Materials and Methods

### 4.1. Study Participants

Patients for this study who had undergone hysterectomy were recruited at the Department of Gynaecology and Gynaecological Oncology of the University Hospital in Bialystok. The study group consisted of 37 patients who had undergone surgery for diagnosed type I endometrial cancer, FIGO stage 1, grade 2 (endometrial cancer group—EC). The control group (C) comprised 17 patients who had undergone surgery for pelvic organ prolapse, matched by age and BMI.

### 4.2. Sample Collection

During the surgical procedure, a tissue sample of the tumor was taken from the uterine cavity in the study group, and a sample of the endometrium was taken from the patients in the control group. None of the patients had undergone chemotherapy or radiation therapy prior to the surgery. Diagnoses were independently confirmed by two pathologists following the standards recommended by the International Federation of Gynaecology and Obstetrics. Tissue samples were immediately placed in liquid nitrogen and then stored at −80 °C until analysis.

Serum was collected by allowing freshly drawn blood to clot, followed by centrifugation at 2000× *g* for 10 min in a refrigerated centrifuge. The resulting supernatant was collected and stored at −80 °C until further analysis. Serum insulin levels were assayed by means of the immunoradiometric method (DiaSource Europe SA, Ottignies-Louvain-la-Neuve, Belgium).

### 4.3. Laboratory Assessments

All the participants underwent clinical examination, anthropometric measurements, and appropriate laboratory tests. Those tests included measuring glucose and insulin concentrations as well as serum triglyceride (TG), total cholesterol (TChol), high-density lipoprotein cholesterol (HDL), and low-density lipoprotein cholesterol (LDL) concentrations. All assessments were conducted after an overnight fast. Plasma glucose concentration was measured by means of an enzymatic method with hexokinase (Cobas c111, Roche Diagnostics Ltd., Basel, Switzerland). Serum TG, TChol, HDL, and LDL concentrations were determined by means of colorimetric methods with a Cobas c111 automatic chemistry analyzer (Roche Diagnostics, Switzerland).

Indirect indices of insulin sensitivity/resistance were examined based on fasting or OGTT plasma glucose and serum insulin concentrations. A homeostasis model assessment of insulin resistance (HOMA-IR) was also calculated. A standard oral glucose tolerance test (OGTT) was performed in patients without a history of diabetes after an overnight fast. Blood samples were collected at 0, 30, 60, and 120 min after a 75 g glucose load.

The body mass index (BMI) was calculated in terms of weight in kilograms divided by height in meters squared.

#### 4.3.1. Sphingolipids Measurement

Sphingolipids were analyzed by means of ultra-high-performance liquid chromatography coupled with tandem mass spectrometry (UHPLC/MS/MS) as recommended by Blachnio-Zabielska et al. [51]. To each sample (tissue homogenates or serum), a mixture of internal standards (ISTD): Sph-d7, SPA-d7, S1P-d7, C15:0-d7-Cer, C16:0-d7-Cer, C18:1-d7-Cer, C18:0-d7-Cer, 17C20:0-Cer, C24:1-d7-Cer, C24-d7-Cer (Avanti Polar Lipids, Alabaster, AL, USA) as well as 2 mL of an extraction solution (isopropanol, water, ethyl acetate, 30:10:60; *v*:*v*:*v*) were added. The samples were vortexed, sonicated, and centrifuged at 4000 rpm, 4 °C for 10 min (Sorvall Legend RT). The supernatants were transferred to new vials and the pellets were re-extracted. The supernatants were combined and evaporated under nitrogen. The dried samples were suspended in LC Solvent B (2 mM Ammonium formate, 0.1% formic acid in methanol) for the purpose of UHPLC/MS/MS analysis. Sphingolipids: Sph (sphingosine), SPA (sphinganine), S1P (sphingosine-1-phosphate), C14:0-Cer, C16:0-Cer, C18:1-Cer, C18:0-Cer, C20:0-Cer, C22:0-Cer, C24:1-Cer, and C24:0-Cer were separated on a reverse-phase Zorbax SB-C8 column 2.1 × 150 mm, 1.8 μm in a binary gradient (1 mM ammonium formate, 0.1% formic acid in water as Solvent A, and 2 mM ammonium formate, 0.1% formic acid in methanol as Solvent B, at a flow rate of 0.4 mL/min). The analyzed sphingolipids were quantified by means of a Sciex Qtrap 6500 + mass spectrometer (SCIEX, Framingham, MA, USA) in a positive electrospray ionization (ESI) (except for S1P that was analyzed in negative mode) with multiple reaction monitoring (MRM) against standard curves constructed for each analyzed compound. The sphingolipid rheostat was calculated by determining the ratio of the S1P [pmol/mg tissue] to total ceramide [pmol/mg tissue] in endometrial tissue [42,52].

#### 4.3.2. Oxidative Status

The total antioxidant capacity (TAC) of the analyzed samples was measured colorimetrically, as recommended by Erel [53], based on the measurement of the ability to neutralize the radical cation ABTS + [2,2-azino-bis-(3-ethylbenzothiazoline-6-sulfonate)] under the influence of antioxidants present in the analyzed samples. Changes in the absorbance of the ABTS + solution were determined at a 660 nm wavelength. TAC levels were calculated using a calibration curve constructed for 6-hydroxy-2,5,7,8-tetramethylchroman-2-carboxylic acid (Trolox). The total oxidant status (TOS) was determined bichromatically (560/800 nm) by means of the colorimetric method described by Erel [19], based on the oxidation of ferrous ions (Fe^2+^) to ferric ions (Fe^3+^) under the influence of oxidants present in the samples. Fe^3+^ ions were detected using xylene orange. The TOS concentration was calculated using a standard curve constructed for hydrogen peroxide and expressed in terms of a micromolar hydrogen peroxide equivalent per liter. The TOS assay was performed in triplicate for each sample.

Oxidative stress index (OSI) was calculated using the following formula [19]:

OSI = [TOS]/[TAC] × 100%

#### 4.3.3. Lipid Peroxidation

Lipid peroxidation was determined by measuring the level of MDA, an end product of lipid peroxidation. MDA content in endometrial tissue was measured using the Lipid Peroxidation (MDA) Assay Kit (Sigma Aldrich, St. Louis, MO, USA, MAK2085), according to the manufacturer’s protocol.

### 4.4. Statistical Analysis

The statistical analysis was performed by means of GraphPad Software’s Prism 9.3.1 (GraphPad Software, La Jolla, CA, USA). Data are presented in terms of medians and interquartile ranges. Differences between the study and control groups were compared by means of the Mann–Whitney U test, and the relationship between variables was examined by means of Pearson correlation. The Wilcoxon signed-rank test was used for comparing sphingolipid concentrations between women with endometrial cancer and healthy controls, and a *p*-value less than 0.05 was considered statistically significant. 

## Figures and Tables

**Table 1:** Clinical characteristics of the groups.

		Control		EC	
n		17		43	
Age (years)		59 (36–82)		64 (39–86)	
Weight		80 (72–116)		83 (50–139)	
BMI (kg/m^2^)		29.76 (20.9–39.21)		32.23 (21.64–54.98)	
Fasting glucose (mg/dL)		108.13 (81–143)		116.97 (84–231)	
Glucose 30′ (mg/dL)		167.23 (82–262)		185.93 (139–307)	
Glucose 60′ (mg/dL)		160.15 (110–221)		202.15 (108–356) *	
Glucose 120′ (mg/dL)		123.61 (44–185)		174.51 (78–340) **	
HOMA–IR		8.89 (0.93–18.07)		18.30 (0.96–48.87)	
Menopause	Premenopausal	50 (45–56)	47.25 (40–54)	(44–57) **	- *
Fertility		2 (0–4)		3(0–8)	
Total cholesterol (mmol/L)		208.8 (138–302)		176.23 (153–213) *	
HDL-cholesterol (mmol/L)		60.40 (34.41–106.93)		56.82 (26.62–83.74)	
LDL-cholesterol (mmol/L)		128.90 (56.6–217.8)		108.72 (73.2–152.3)	
Triglycerides (mmol/L)		145.15 (60–280)		122 (41–267)	
MDA (mmol/mg)		1.96 (1.88–2.00)		3.75 (3.56–3.92) ****	

Data are presented as medians (interquartile range) and the differences between groups were compared by Mann–Whitney U test; * *p* < 0.05, ** *p* < 0.01, **** *p* < 0.0001 indicate significant differences from the controls. *n*—number, BMI—Body Mass Index, HOMA-IR—Homeostasis Model Assessment of Insulin Resistance, HDL—High-density lipoprotein, LDL—Low-density lipoprotein, MDA—malondialdehyde, end product of lipid peroxidation.

**Table 2:** The concentration of sphingolipids in the tissues of the two study groups.

	Control	EC	*p*-Value
	Me (Q1–Q3) [pmol/mg]	Me (Q1–Q3) [pmol/mg]	
Sph	1.67 (1.37–2.34)	5.49 (4.97–6.09) ****	<0.0001
SPA	0.48 (0.39–0.71)	2.14 (1.79–2.46) ****	<0.0001
S1P	0.15 (0.13–0.16)	0.21 (0.17–0.31) ****	<0.0001
C14:0 Cer	0.17 (0.21–0.13)	0.92 (0.81–1.09) ****	<0.0001
C16:0 Cer	14.85 (12.28–17.34)	46.32 (36.17–54.42) ****	<0.0001
C18:1 Cer	0.08 (0.07–0.09)	0.26 (0.23–0.41) ****	<0.0001
C18:0 Cer	3.20 (2.39–3.39)	4.76 (4.07–5.83) ****	<0.0001
C20:0 Cer	0.65 (0.56–0.72)	1.10 (0.90–1.41) ****	<0.0001
C22:0 Cer	2.62 (1.94–3.07)	5.20 (4.30–6.03) ****	<0.0001
C24:1 Cer	9.05 (8.68–13.96)	14.96 (13.30–16.77) ****	<0.0001
C24:0 Cer	6.30 (5.56–7.03)	11.71 (9.74–12.93) ****	<0.0001
Cer Total	36.99 (32.22–44.63)	82.69 (70.58–96.02) ****	<0.0001

Data are presented as medians (interquartile range). Analysis was performed with the Mann–Whitney U test; **** *p* < 0.0001 indicate significant differences from the controls. Sph—Sphingosine, SPA—Sphinganine, S1P—Sphingosine-1-phosphate, C14:0 Cer—Ceramide with a 14 carbon acyl chain length, C16:0 Cer—Ceramide with a 16 carbon acyl chain length, C18:1 Cer—Ceramide with an 18 carbon acyl chain length, C18:0 Cer—Ceramide with an 18 carbon acyl chain length, C20:0 Cer—Ceramide with a 20 carbon acyl chain length, C22:0 Cer—Ceramide with a 22 carbon acyl chain length, C24:1 Cer—Ceramide with a 24 carbon acyl chain length, C24:0 Cer—Ceramide with a 24 carbon acyl chain length, Cer Total—Total ceramide concentration in the sample.

**Table 3:** The concentration of sphingolipids in serum of the two study groups.

	Control	EC
	Me (Q1–Q3) [pmol/mL]	Me (Q1–Q3) [pmol/mL]
Sph	6.01 (5.02–7.51)	6.36 (4.88–8.12)
SPA	3.54 (2.97–5.26)	4.18 (3.29–5.69)
S1P	690.74 (622.36–812.05)	719.0 (605.8–798.5)
C14:0 Cer	17.40 (14.77–21.47)	18.42 (16.52–20.23)
C16:0 Cer	198.3 (165.1–259.6)	186.8 (156.6–225.4)
C18:1 Cer	9.82 (8.99–10.96)	10.83 (8.65–14.40)
C18:0 Cer	98.1 (84.2–123.7)	95.6 (80.5–124.2)
C20:0 Cer	130.1 (95.7–177.6)	135.5 (114.7–165.6)
C22:0 Cer	496.8 (343.4–549.4)	429.2 (338.4–496.3)
C24:1 Cer	934.3 (666.5–1050.1)	837.5 (700.1–1144.2)
C24:0 Cer	2506.2 (2234.5–3269.0)	2644.3 (1894.4–2915.1)
Cer Total	4510.9 (3787.9–5227.0)	4426.6 (3580.2–4830.8)

Data are presented as medians (interquartile range). Analysis was performed with the Mann–Whitney U test.

**Table 4:** Correlations between TOS, TAC, and OSI with sphingolipids.

	TOS	TAC	OSI
Sph	r = 0.5074 *p* = 0.0135 *	r = −0.0352 *p* = 0.8733	r = 0.3999 *p* = 0.0587
SPA	r = 0.5517 *p* = 0.0064 **	r = −0.1447 *p* = 0.5101	r = 0.5427 *p* = 0.0075 **
S1P	r = 0.1732 *p* = 0.4295	r = −0.2284 *p* = 0.2945	r = 0.2100 *p* = 0.3361
C14:0 Cer	r = 0.4557 *p* = 0.0289 *	r = −0.1489 *p* = 0.4976	r = 0.4257 *p* = 0.0428 *
C16:0 Cer	r = 0.5212 *p* = 0.0108 *	r = −0.1630 *p* = 0.4573	r = 0.4935 *p* = 0.0167 *
C18:1 Cer	r = 0.4139 *p* = 0.0496 *	r = −0,1216 *p* = 0.5805	r = 0.3799 *p* = 0.0738
C18:0 Cer	r = 0.3102 *p* = 0.1498	r = −0.1820 *p* = 0.4058	r = 0.3582 *p* = 0.0933
C20:0 Cer	r = 0.5010 *p* = 0.0149 *	r = 0.0736 *p* = 0.7386	r = 0.2845 *p* = 0.1883
C22:0 Cer	r = 0.6415 *p* = 0.0010 ***	r = −0.1616 *p* = 0.4612	r = 0.5822 *p* = 0.0036 **
C24:1 Cer	r = 0.2332 *p* = 0.2843	r = −0.2166 *p* = 0.3208	r = 0.4353 *p* = 0.0379 *
C24:0 Cer	r = 0.5518 *p* = 0.0063 **	r = −0.1467 *p* = 0.5042	r = 0.5084 *p* = 0.0132 *
Total Cer	r = 0.5214 *p* = 0.0107 *	*p* = 0.4194 r = −0.1769	r = 0.5226 *p* = 0.0105 *

Data are presented as Pearson correlation coefficient; statistical significance: * *p* < 0.05, ** *p* < 0.01, *** *p* < 0.001. Sph—Sphingosine, SPA—Sphinganine, S1P—Sphingosine-1-phosphate, C14:0-Cer—Ceramide with a 14 carbon acyl chain length, C16:0-Cer—Ceramide with a 16 carbon acyl chain length, C18:1-Cer—Ceramide with an 18 carbon acyl chain length, C18:0-Cer—Ceramide with an 18 carbon chain length, C20:0-Cer—Ceramide with a 20 carbon acyl chain length, C22:0-Cer—Ceramide with a 22 carbon acyl chain length, C24:1-Cer—Ceramide with a 24 carbon acyl chain length, C24:0-Cer—Ceramide with a 24 carbon acyl chain length, Total Cer—Total ceramide concentration in the sample. *p*-Value—Probability, r—Correlation coefficient.

## Data Availability

Data generated and/or analyzed in the current study are available at the following link: https://osf.io/hda42/?view_only=a07f7d2f3c724b2ca0b576c8cd387730 (accessed on 15 September 2024).
